# Relationship between oxidative balance score and health-related quality of life in Korean adults

**DOI:** 10.1371/journal.pone.0330837

**Published:** 2025-09-04

**Authors:** Jinheum Kim, Subin Seong, Eunjeong Cha

**Affiliations:** 1 Department of Applied Statistics, University of Suwon, Hwaseong, Korea; 2 Department of Data Science, University of Suwon, Hwaseong, Korea; 3 Department of Nursing Science, University of Suwon, Hwaseong, Korea

## Abstract

This study aimed to examine the relationship between the oxidative balance score (OBS) and health-related quality of life (HRQoL) among Korean adults. We analyzed data from 4,381 individuals aged 19 years or older from the 2021 Korea National Health and Nutrition Examination Survey. The OBS was calculated based on nutrients and lifestyle factors, including five pro-oxidants and ten antioxidants. We fitted logistic quantile regression models to investigate the relationship between OBS and HRQoL. We found that the distribution of each OBS component differed significantly between men and women, except for retinol and physical activity. Men exhibited a more favorable oxidative balance in terms of antioxidant components, whereas women demonstrated a more favorable oxidative balance in relation to pro-oxidant components. This distinction highlights that although men had higher scores for antioxidant intake, women showed lower exposure to pro-oxidants. Both aspects contribute differently to overall oxidative balance and may have distinct implications for HRQoL. The logistic quantile regression model indicated that each 1-point increase in OBS was associated with a 1.02-fold increase in the median HRQoL Instrument with eight items (HINT-8) index (*p* = 0.006). The median HINT-8 index was 1.28 times higher in men compared to women (*p* < 0.001) and 1.20 times higher among individuals in the first and second income quintiles compared to those in the third and fourth quintiles (*p* = 0.004). This study confirmed the positive influence of OBS on HRQoL. Therefore, OBS could be used to assess oxidative stress risk and to develop tailored interventions aimed at improving health.

## Introduction

Reactive oxygen species (ROS) are chemically reactive molecules containing oxygen and are often referred to as “free radicals” because they typically possess at least one unpaired electron in their outer orbitals [1]. Oxygen-free radicals, or more generally ROS, are products of normal cellular metabolism [2]. They are recognized for playing a dual role, as they can be both harmful and beneficial to living organisms [3]. Low to intermediate levels of ROS are necessary for maintaining essential physiological functions, preserving redox homeostasis, and regulating key transcription factors. In contrast, excessive levels of ROS disrupt redox homeostasis, leading to oxidative stress (OS) and ROS-mediated damage to essential biomolecules, including deoxyribonucleic acid, proteins, and cell membranes [4].

OS was previously defined as an imbalance between oxidants and antioxidants, which can have harmful effects on the body [5]. These harmful effects have led to OS being recognized as central to the pathophysiology of many disorders [6]. OS leads to the development of various diseases, including atherosclerosis, ischemic heart disease, liver disorders, diabetes, and carcinogenesis [7]. Cells have developed a complex antioxidant system composed of enzymatic and low molecular antioxidants, whose coordinated action helps maintain oxidative balance [8]. This complex antioxidant defense system protects cells from ROS-induced toxicity [9]. Antioxidants are divided into antioxidant enzymes and low molecular weight antioxidants. The primary antioxidant enzymes include superoxide dismutase, catalase, and glutathione peroxidase, while the key low molecular weight antioxidants include vitamin C, vitamin E, carotenoids, flavonoids, and glutathione [10].

Pro-oxidants can induce OS by generating ROS or by impairing the activity of antioxidants [11]. Various pro-oxidant and antioxidant factors were identified in a previous study [12]. High intake of certain dietary nutrients, including vitamin C, vitamin E, and carotenoids, may exert protective effects against OS, whereas pro-oxidant factors, such as smoking and high intake of fat and iron, can generate ROS and accelerate OS-related cellular damage [12–14]. Goodman et al. [15] found that the oxidative balance score (OBS), a semi-quantitative measure of OS, was associated with both prostate cancer and colorectal adenoma, but not with the individual components of the OBS. A composite measure of multiple pro-oxidant and antioxidant exposures may provide a more accurate indicator of an individual’s OS burden [16]. The OBS has been validated in several studies on cancer, chronic kidney disease, and cardiovascular disease [17–19], and has proven valuable for chronic disease (CD) research in epidemiological studies [20]. In addition, previous studies have found that OBS is inversely associated with various diseases, including metabolic syndrome, respiratory disorders, cardiovascular disease, and type 2 diabetes [21–23]. OBS assesses the overall balance of an individual’s pro-oxidants and antioxidants [15], and quantifies the pro-oxidant and antioxidant components of dietary and lifestyle factors [19].

In a systematic literature review on OBS, most studies suggested that excessive OS, indicated by a lower OBS, has deleterious effects on health [24]. In a study examining the relationship between OBS and health-related quality of life (HRQoL) in patients with osteoarthritis, no association was found between osteoarthritis and OBS; however, HRQoL and OBS were positively associated [12]. HRQoL is a multidimensional concept that refers to functioning and perceived well-being across the physical, mental, and social domains of life [25]. It is considered an essential indicator of the overall health status of adults [26,27]. Therefore, although OBS can serve as a health management indicator by demonstrating its association with HRQoL in the general adult population, research in this area remains limited.

The EuroQol 5-Dimension 3-Level (EQ-5D-3L) has been used to measure HRQoL since 2005, when it was introduced in the Korea National Health and Nutrition Examination Survey (KNHANES) conducted on the general Korean population. However, concerns were raised regarding the ceiling effect and potential cultural differences when applied to Korean respondents. Therefore, as an alternative, a general HRQoL measurement tool based on preference—the “Health-related Quality of Life Instrument with eight items (HINT-8)—was developed, which offers advantages for comparing HRQoL in the general population [28,29]. This study used the HINT-8 to measure the overall health status of the research participants.

When identifying risk factors for a response variable, linear regression models (LMs) are commonly used if the response variable is continuous and unconstrained in its possible values. However, if the continuous response variable is restricted to a specific range, predicted values from the LM may fall outside that range. One approach to address this issue is to convert the continuous response variable into a binary variable based on a predefined threshold, and then apply a logistic regression model. In this approach, rather than modeling the expected value of the continuous response variable Y given the risk factors, the focus shifts to modeling the expected value of the binary-transformed variable $Y^{*}$. Since the expected value of $Y^{*}$ represents a probability p, it is naturally constrained between 0 and 1. To address this constraint, logistic regression applies a transformation that maps these probabilities to an unrestricted scale. When the distribution of Y is asymmetric, a regression model that utilizes a robust measure, such as the median, which is less sensitive to outliers and error distribution misspecification, may be more useful than one based on the mean of Y [30]. In addition to the median, regression models can be fitted for other quantiles; such models are referred to as quantile regression models (QM). QM has the advantage of predicting not only the central tendency of the response variable but also its relative positions across the entire distribution. However, when the response variable is constrained within a specific range, the predicted values, similar to those in LMs, may fall outside the permissible range. To address this issue, a logistic quantile regression model (LQM) was proposed, in which Y is linearly transformed and then mapped using a logit function, rather than mapping the probability p, as described in the statistical analysis section [31–34]. Unlike LM or QM, this model ensures that the predicted values always remain within the range of Y.

This study aimed to elucidate the relationship between the OBS and HRQoL using the LQM and to establish a foundation for employing OBS as a key element in strategies aimed at promoting health and preventing disease.

## Materials and methods

### Participants

**Fig 1:**
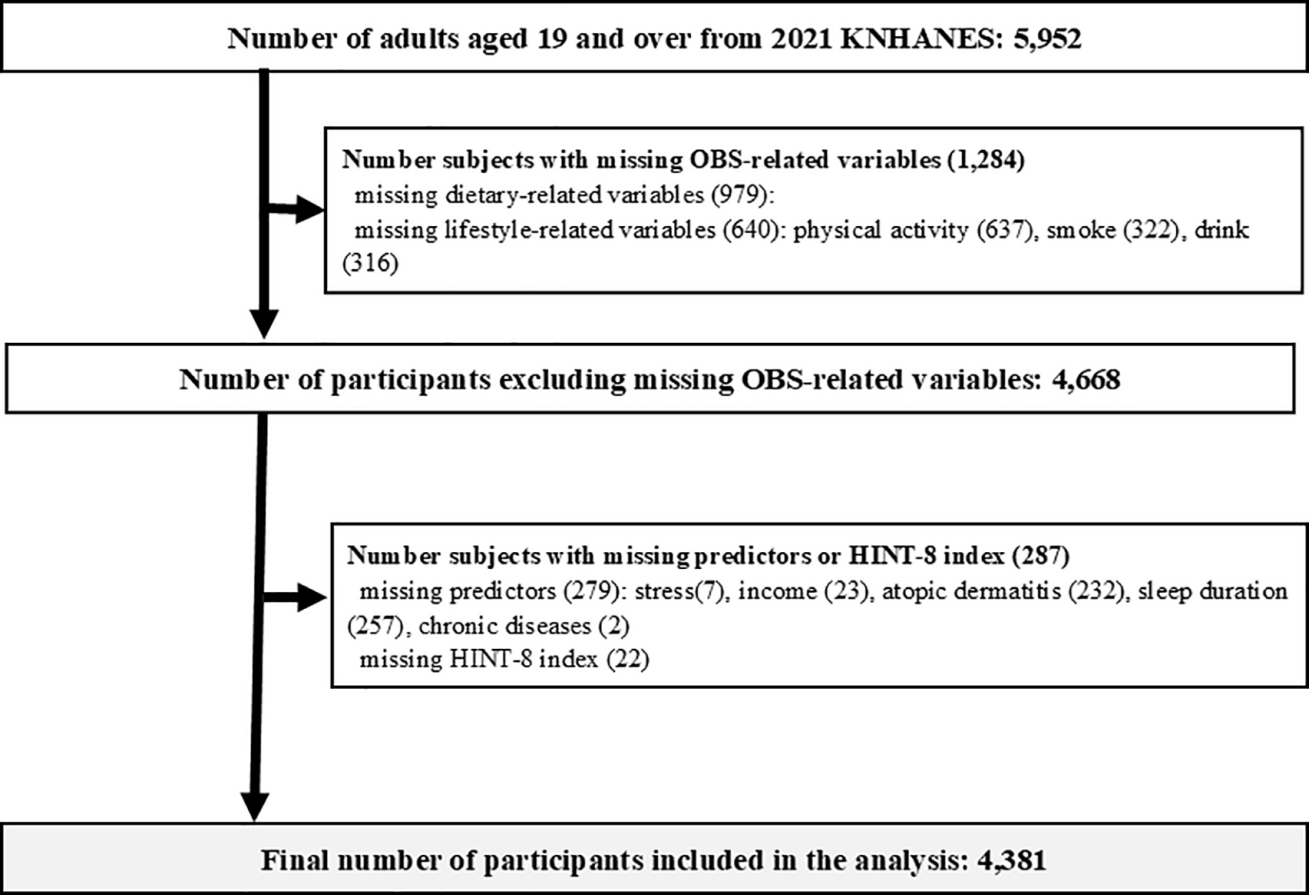
A flowchart detailing the process of study participant selection.

For the survey design of the 8th KNHANES (2019–2021), the most recent available Population and Housing Census data from 2016 were used as the primary survey base, as these were the latest data available when the sampling plan was developed. To ensure that participants adequately represented the target population, which includes all individuals aged ≥ 1 year residing in the Republic of Korea, the survey base was stratified by province and housing type (i.e., general housing, apartments). A two-stage stratified cluster sampling method was applied, with survey districts and households selected as the primary and secondary respondents, respectively. We analyzed secondary data from the 8th KNHANES [35], a cross-sectional survey conducted by the Korea Disease Control and Prevention Agency, involving household member verification, health questionnaires, medical examinations, and nutritional assessments within the Korean target population. Among the 5,952 adults aged ≥ 19 years in the 2021 KNHANES, 1,284 individuals with at least one missing value in the variables used to define the OBS were excluded, reducing the sample size to 4,668. An additional 287 individuals with missing values in predictors or the HINT-8 index were excluded, resulting in a final analytical sample of 4,381 participants. Fig 1 presents a detailed flowchart illustrating this inclusion and exclusion process.

KNHANES-VIII was approved by the Institutional Review Board (IRB) of the Korea Centers for Disease Control and Prevention (KCDC) (approval number: 2018-01-03-C-A) and was conducted in accordance with the principles of the Declaration of Helsinki. Written informed consent was obtained from all participants before their participation in the survey. Additionally, **t**his study was approved by the Institutional Review Board of the authors’ institution(s) (approval number: USW IRB/ 2410-045-01 and Aug 2024).

### Measurements of OS and HRQoL

#### OBS for measuring OS.

The OBS was calculated based on previously established relationships between OS, nutrient intake, and lifestyle behaviors [12–14,19,26]. A total of 15 components were included: 12 dietary factors and 3 lifestyle factors. Among them, 10 were classified as antioxidants and 5 as pro-oxidants.

Dietary factors included vitamins C, D, E, and B_9_; β-carotene; fiber; zinc; retinol; calcium; iron; saturated fatty acids (SFA); and polyunsaturated fatty acids (PUFA). For antioxidants, the first through third tertiles were assigned 0–2 points, respectively, whereas the scoring for pro-oxidants (iron, SFA and PUFA) was reversed (0 points for the third tertile and 2 points for the first tertile).

Lifestyle factors included physical activity, alcohol consumption, and smoking status. Physical activity was categorized by total weekly metabolic equivalent of task (MET)-hours as low (<7.5 MET-h/week), moderate (7.5–30 MET-h/week), and high (>30 MET-h/week), and assigned scores of 0, 1, and 2, respectively [36]. Alcohol consumption was grouped into heavy drinkers (≥30 g/day for men, ≥ 20 g/day for women), non-heavy drinkers (<30 g/day for men, < 20 g/day for women), and non-drinkers, with scores of 0, 1, and 2, respectively [22,44]. Smoking status was categorized as current (0), former (1), and never (2) [18].

Therefore, the total OBS ranged from 0 to 30, with higher scores indicating a more favorable oxidative balance.

#### HINT-8 index for measuring HRQoL.

HRQoL was measured using the HINT-8 assessment instrument developed by the Korea Disease Control and Prevention Agency. HINT-8 is a preference-based tool in which respondents answer pre-described items, and item-specific quality weights are derived based on the evaluator’s preferences. The tool is particularly useful for comparing HRQoL in the general population, conducting economic evaluations, and assessing health-adjusted life expectancy. HINT-8 measures HRQoL in South Korea across four domains—physical, social, mental, and positive—using eight items: climbing stairs (CL), pain (PA), vitality (VI), working (WO), depression (DE), memory (ME), sleep (SL), and happiness (HA).

The items are rated using a 4-point Likert scale (1 = always good; 2 = usually good; 3 = sometimes good; 4 = poor) [28]. These ratings are then incorporated into the HINT-8 index, which is defined as follows [29]:

HINT-8 index = 1 - 0.073 - 0.018 × CL2 - 0.072 × CL3 - 0.122 × CL4 - 0.055 × PA2 - 0.116 × PA3 - 0.188 × PA4 - 0.019 × VI23 - 0.070 × VI4 - 0.004 × WO2 - 0.028 × WO3 - 0.036 × WO4 - 0.012 × DE2 - 0.044 × DE3 - 0.098 × DE4 - 0.014 × ME2 - 0.058 × ME3 - 0.109 × ME4 - 0.020 × SL3 - 0.090 × SL4 - 0.014 × HA2 - 0.068 × HA3 - 0.082 × HA4,

Binary variables CL2, CL3, and CL4 are assigned a value of 1 if the variable CL equals 2, 3, or 4, respectively, and 0 otherwise. Similarly, PA2, PA3, and PA4 follow the same logic for PA; WO2, WO3, and WO4 for WO; DE2, DE3, and DE4 for DE; ME2, ME3, and ME4 for ME; and HA2, HA3, and HA4 for HA. The variable VI23 is set to 1 if VI equals 2 or 3 and 0 otherwise, whereas VI4 is 1 if VI equals 4 and 0 otherwise. Finally, SL3 and SL4 are assigned a value of 1 if SL equals 3 or 4, respectively, and 0 otherwise.

Therefore, a higher HINT-8 index indicates better HRQoL. The EQ-5D-3L includes 243 (=3^5^) potential health states, whereas the HINT-8 encompasses over 65,536 (=4^8^) possible health states. Consequently, the HINT-8 index offers a broader range of potential values, a more balanced distribution, and a reduced ceiling effect, even when respondents report no issues across all items.

### Covariates

In our study, covariates included factors that were previously demonstrated or hypothesized to be associated with HRQoL or OBS. These covariates included socio-demographic factors, chronic diseases (CDs), atopic dermatitis (AD), sleep duration, stress status, and abdominal obesity based on waist circumference. Socio-demographic covariates included age, gender, education level, household type, and household income. CDs included hypertension, hyperlipidemia, cardiovascular disease, diabetes, and arthritis, and were classified as “yes” or “no.” Sleep duration was classified based on the recommended sleep duration of 7–9 h [37]. Stress status was assessed using the question, “How much stress do you usually feel in your daily life?” Responses were categorized as “no” if the response was “rarely feel stress” or “feel a little stress,” and as “yes” if the answer was “feel a lot of stress” or “feel very much stress.” Abdominal obesity was defined as “yes” if the waist circumference was ≥ 90 cm for men or ≥ 85 cm for women, and “no” otherwise.

### Statistical analysis

First, we summarized the distribution of each OBS component by gender and tested for statistical significance using the Rao–Scott chi-square test [38] to assess whether the distributions for men and women differed, accounting for individual weights determined by the sampling design.

Second, we examined the association between the covariates, excluding gender, and both the OBS and the HINT-8 index. For covariates with two categories, t-tests were performed, and for those with three or more categories, F-tests were conducted. As previously mentioned, all analyses accounted for individual weights.

Third, we utilized the LQM to investigate the relationship between OBS and HRQoL. Bottai et al.’s [31] LQM can be summarized as follows: Let the random variable *Y* be an outcome with a value in interval $\left[c_{1},c_{2}\right]\subset (-\infty ,\infty )(c_{1}<c_{2})$, and let $X=(1,x_{1},x_{2},\ldots ,x_{s})\prime$ be a (s+1)-dimensional vector of covariates. Let p∈(0,1). When X=x is given, let the (100×p) percentile of *Y* be $Q_{p}\left(y|x\right)$. That is, for a given p∈(0,1), $P(Y\leq Q_{p}\left(y|x)|X=x\right)=p$. A regression model that links $Q_{p}(y|x)$ and a linear predictor $x^{\prime }\beta_{p}$ with an identity function is called the QM of Y. That is, for a given p∈(0,1),


$$Q_{p}\left(y|x\right)=x^{\prime }\beta_{p}+\epsilon ,$$ (1)

where P(ϵ≤0)=p and $\begin{array}{c} \beta_{p}=(\beta_{p0}{,\;\beta }_{p1},\beta_{p2},\ldots ,\beta_{ps}{)}^{\prime } \end{array}$ represents a vector of regression coefficients. However, a limitation of this model is that while outcome values are restricted to the interval $\left[c_{1},c_{2}\right]$, the estimated linear predictor spans (−∞,∞), meaning the predicted value of $Q_{p}(y|x)$ does not necessarily belong to the interval $\left[c_{1},c_{2}\right]$. Let the response variable y, constrained to the interval $\left[c_{1},c_{2}\right]$, be transformed as follows:


$$y^{*}=\frac{y-c_{1}}{c_{2}-c_{1}},y\in \left[c_{1},c_{2}\right].$$ (2)

When linearly converted, the converted value falls in the interval (0,1). However, if $y=c_{1}$, then $y^{*}=0$, and the odds of $y^{*}$ is 0, so the logarithm of $y^{*}$ is not defined. Similarly, if $y=c_{2}$, then $y^{*}=1$, and the odds of $y^{*}$ is not defined. Therefore, Bottai et al. [31] substitute $c_{1}$ with $c_{1}-\delta$ and $c_{2}$ with $c_{2}+\delta$ in equation (2), where δ is a very small constant greater than 0. To ensure the logarithm of $y^{*}$ at the end of interval $\left[c_{1},c_{2}\right]$, equation (2) can be expressed as follows:


$$y^{*}=\frac{y-c_{1}+\delta }{c_{2}-c_{1}+2\delta },y\in \left[c_{1},c_{2}\right]$$ (3)

Given *x,* let the (100×p) percentile of $Y^{*}$ be $Q_{p}\left(y^{*}|x\right)$. That is, $P(Y^{*}\leq Q_{p}\left(y^{*}|x)|X=x\right)=p$ for p∈(0,1). A regression model that links $Q_{p}\left(y^{*}|x\right)$ and a linear predictor $x^{\prime }\beta_{p}$ using a logit function, specifically, the LQM, which is analogous to a logistic regression model, can be defined as follows:


$$\text{log}\left(\frac{Q_{p}\left(y^{*}|x\right)-c_{1}+\delta }{c_{2}+\delta -Q_{p}(y^{*}|x)}\right)=x^{\prime }\beta_{p}.$$ (4)

For a given p, let ${\hat{\beta }}_{p}$ be the maximum likelihood estimate of $\beta_{p}$ obtained from the model (4). Furthermore, plugging ${\hat{\beta }}_{p}$ into (4) and inverting (4), we can define the estimate of $Q_{p}\left(y^{*}|x\right)$ as follows:


$${\hat{Q}}_{p}\left(y^{*}|x\right)=\frac{\left(c_{2}+\delta \right)\times \text{exp}\left(x^{\prime }{\hat{\beta }}_{p}\right)+c_{1}-\delta }{1+\text{exp}\left(x^{\prime }{\hat{\beta }}_{p}\right)}.$$

The Surveyfreq procedure in SAS version 9.4 (SAS Institute Inc., Cary, NC, USA) was used to perform the Rao−Scott chi-square tests. For mean comparisons, t-tests and F-tests were conducted using the Surveymeans and Surveyreg procedures, respectively, in SAS version 9.4 (SAS Institute Inc., Cary, NC, USA). The LQM model was estimated using the Log.lqr function from the lqr package in R (Version 4.3.3; R Core Team, 2024). All models controlled for a set of covariates—including socio-demographic, health-related, and behavioral/psychosocial factors (e.g., stress and sleep duration) factors—selected based on prior evidence of potential confounding. These covariates were included to isolate the independent effect of the OBS on HRQoL.

## Results

The gender-specific distribution of each of the 15 components comprising the OBS is presented in Table 1, along with the results of the homogeneity tests between men and women. The results in Table 1 indicate that, except for retinol and physical activity, the distributions of all components differed significantly between men and women. Notably, men scored higher than women on antioxidant components, whereas women scored higher on pro-oxidant components.

**Table 1:** Frequency of each component of the oxidative balance score (OBS) by gender.

				N (%)		
Factor	Type	Component	Points	Male	Female	p-value
Pro-oxidant	Dietary	SFA	0	796 (48.4)	694 (30.9)	< 0.001
			1	624 (31.0)	862 (34.8)	
			2	459 (20.6)	946 (34.3)	
		Iron	0	828 (45.8)	670 (26.8)	< 0.001
			1	627 (32.7)	850 (34.1)	
			2	424 (21.5)	982 (39.1)	
		PUFA	0	795 (46.0)	708 (30.3)	< 0.001
			1	619 (32.3)	854 (35.4)	
			2	465 (21.7)	940 (34.3)	
	Lifestyle	Smoking	0	528 (30.4)	114 (5.2)	< 0.001
			1	799 (38.4)	126 (5.2)	
			2	552 (31.2)	2262 (89.6)	
		Alcohol	0	238 (13.8)	89 (4.1)	< 0.001
		consumption	1	1186 (65.9)	1366 (60.0)	
			2	455 (20.3)	1047 (35.9)	
Antioxidant	Dietary	Vitamin C	0	567 (30.1)	850 (34.7)	0.011
			1	666 (36.2)	802 (32.9)	
			2	646 (33.7)	850 (32.4)	
		Vitamin E	0	425 (20.7)	977 (36.9)	< 0.001
			1	618 (32.8)	868 (36.6)	
			2	836 (46.5)	657 (26.5)	
		β-carotene	0	520 (28.7)	923 (37.7)	< 0.001
			1	657 (34.8)	793 (32.0)	
			2	702 (36.5)	786 (30.3)	
		Fiber	0	458 (26.3)	968 (42.4)	< 0.001
			1	620 (33.7)	825 (32.6)	
			2	801 (40.0)	709 (25.0)	
		Zinc	0	344 (17.8)	1068 (43.6)	< 0.001
			1	627 (31.8)	856 (33.5)	
			2	908 (50.4)	578 (22.9)	
		Vitamin B_9_	0	458 (25.5)	962 (40.4)	< 0.001
			1	625 (34.1)	832 (33.1)	
			2	796 (40.4)	708 (26.5)	
		Retinol	0	591 (26.8)	813 (28.4)	0.328
			1	636 (34.5)	842 (35.4)	
			2	652 (38.7)	847 (36.2)	
		Vitamin D	0	545 (28.4)	903 (35.7)	< 0.001
			1	637 (35.1)	814 (33.4)	
			2	697 (36.5)	785 (30.9)	
		Calcium	0	482 (25.6)	933 (37.9)	< 0.001
			1	639 (33.9)	826 (33.7)	
			2	758 (40.5)	743 (28.4)	
	Lifestyle	Physical	0	491 (24.8)	674 (24.9)	0.206
		activity	1	764 (40.3)	1044 (42.7)	
			2	622 (34.9)	778 (32.4)	

SFA: saturated fatty acids, PUFA: polyunsaturated fatty acids.

The results of assessing the statistical differences across the categories of each covariate in the OBS and HINT-8 index are presented in Table 2. Socio-demographic factors that demonstrated differences in the OBS included age and type of household, while the health-related covariates were AD, CDs, sleep duration, and stress status. All socio-demographic and health-related covariates, except for AD, demonstrated differences in the HINT-8 index across categories. Specifically, participants aged 65 years and older had a higher OBS than those in the 19–39 and 40–64 age groups, and participants in multiple-person households had a higher OBS than those in single-person households. Participants with CDs had a higher OBS than those without CDs; those without AD had a higher OBS than those with AD; and participants with less than 7 h of sleep duration (indicating slightly insufficient sleep) had a higher OBS than those without this sleep deficiency; and participants who did not report feeling stressed had a higher OBS compared to those who did.

**Table 2:** Association between each covariate and the oxidative balance score (OBS) and the Health-related Quality of Life Instrument with eight items (HINT-8) index, conducted while accounting for individual weight.

		OBS		HINT-8 index×10	
Variable	Category	M ± SE	p-value	M ± SE	p-value
Total		15.7 ± 0.09	–	8.1 ± 0.02	–
Sex	Male	15.7 ± 0.11	0.502	8.3 ± 0.03	<0.001
	Female	15.7 ± 0.11		7.9 ± 0.02	
Age, years	19–39	14.6 ± 0.13	<0.001	8.4 ± 0.03	<0.001
	40–64	16.2 ± 0.11		8.1 ± 0.02	
	≥65	16.3 ± 0.15		7.6 ± 0.04	
Education	Elementary	15.5 ± 0.16	0.294	7.5 ± 0.06	<0.001
	Middle to high	15.7 ± 0.12		8.1 ± 0.03	
	College and above	15.7 ± 0.11		8.3 ± 0.02	
Income	1^st^ or 2^nd^ quartile	15.6 ± 0.12	0.262	7.8 ± 0.04	<0.001
	3^rd^ or 4^th^ quartile	15.8 ± 0.11		8.3 ± 0.02	
Single-person	No	15.8 ± 0.09	0.009	8.2 ± 0.02	<0.001
household	Yes	15.2 ± 0.19		7.8 ± 0.06	
Chronic diseases	No	15.5 ± 0.13	0.054	8.2 ± 0.02	<0.001
	Yes	15.8 ± 0.09		8.0 ± 0.03	
Atopic dermatitis	No	15.7 ± 0.09	0.012	8.1 ± 0.02	0.775
	Yes	14.5 ± 0.50		8.2 ± 0.12	
Sleep duration, h	<7	15.9 ± 0.12	0.007	8.0 ± 0.03	<0.001
	7–9	15.7 ± 0.12		8.2 ± 0.02	
	>9	14.7 ± 0.34		7.9 ± 0.09	
Stress	No	15.9 ± 0.10	<0.001	8.3 ± 0.02	<0.001
	Yes	15.1 ± 0.13		7.6 ± 0.03	
Abdominal	No	15.7 ± 0.10	0.336	8.2 ± 0.02	<0.001
obesity	Yes	15.6 ± 0.12		8.0 ± 0.03	

M: mean, SE: standard error of the mean.

Table 3 presents the estimated odds ratios and their 95% confidence intervals for the regression parameters at *p* = 0.05, 0.25, 0.5, and 0.75 using $c_{1}=0.132,\;c_{2}=1,$ and δ=0.01 in the LQM. Due to space limitations, the statistical significance of the regression coefficients is represented by superscript symbols corresponding to ranges of p-values, rather than listing exact p-values. The superscripts “a”, “b”, “c”, and “d” indicate that the p-value is less than 0.001, 0.01, 0.05, and 0.1, respectively. Focusing on the median HINT-8 index (p=0.5), the LQM demonstrated that a 1-point increase in the OBS resulted in a 1.02-fold increase in the median HINT-8 index (*p* = 0.006). Therefore, the OBS positively influences median HRQoL. Additionally, the OBS improved other quantiles, including the 5th, 25th, and 75th, suggesting that an increase in OBS shifts the distribution of the HINT-8 index to the right.

**Table 3:** Estimated odds ratios and their 95% confidence intervals for regression parameters based on the logistic quantile regression model, along with summary statistics of estimated quantiles.

		Quantile			
Variable	Category	$Q_{0.05}$	$Q_{0.25}$	$Q_{0.5}$	$Q_{0.75}$
OBS	–	1.04^a^	1.02^b^	1.01^c^	1.03^a^
		(1.03,1.04)	(1.01,1.03)	(1.00,1.03)	(1.01,1.04)
Sex	Male				
	Female	1.03^d^	0.85^a^	0.81^a^	0.66^a^
		(1.00,1.07)	(0.79,0.92)	(0.73,0.89)	(0.60,0.73)
Age, years	19-39				
	40–64	0.63^a^	0.79^a^	0.80^b^	0.73^a^
		(0.59,0.66)	(0.71,0.89)	(0.69,0.92)	(0.64,0.84)
	≥65	0.53^a^	0.66^a^	0.68^a^	0.58^a^
		(0.50,0.56)	(0.57,0.76)	(0.57,0.82)	(0.49,0.69)
Education	Elementary				
	Middle to high	1.56^a^	1.30^a^	1.15^d^	0.89^d^
		(1.49,1.64)	(1.17,1.45)	(0.99,1.33)	(0.78,1.01)
	College and above	1.87^a^	1.39^a^	1.17^d^	0.83^c^
		(1.77,1.97)	(1.23,1.58)	(0.98,1.39)	(0.70,0.97)
Income	1^st^ or 2^nd^ quintile				
	3^rd^ or 4^th^ quintile	1.19^a^	1.18^a^	1.19^b^	1.37^a^
		(1.14,1.24)	(1.07,1.29)	(1.05,1.34)	(1.23,1.52)
Single-person	No				
household	Yes	0.74^a^	0.87^b^	0.91	0.86^c^
		(0.71,0.78)	(0.79,0.97)	(0.79,1.04)	(0.75,0.98)
Chronic disease	No				
	Yes	1.20^a^	1.00	1.01	1.08
		(1.15,1.24)	(0.91,1.11)	(0.88,1.15)	(0.95,1.21)
Atopic dermatitis	No				
	Yes	1.07	0.99	0.90	0.71
		(0.91,1.25)	(0.66,1.49)	(0.53,1.53)	(0.44,1.16)
Sleep duration, h	< 7				
	7–9	0.85^a^	1.03	1.04	1.09^d^
		(0.82,0.89)	(0.95,1.12)	(0.94,1.17)	(0.99,1.20)
	> 9	1.02	0.98	0.97	1.04
		(0.93,1.12)	(0.81,1.19)	(0.77,1.23)	(0.85,1.28)
Stress	No				
	Yes	0.47^a^ (0.45,0.49)	0.56^a^ (0.51,0.62)	0.56^a^ (0.48,0.64)	0.40^a^ (0.35,0.45)
Abdominal	No				
obesity	Yes	0.93^a^ (0.89,0.97)	0.94 (0.86,1.03)	0.92 (0.82,1.04)	0.84^b^ (0.76,0.94)
Estimated	Minimum	0.24	0.47	0.61	0.68
quantiles	Median	0.58	0.74	0.83	0.92
of HINT-8	Mean	0.57	0.73	0.81	0.90
index	Maximum	0.82	0.86	0.91	0.97

The superscripts “a”, “b”, “c”, or “d” indicate that the p-value is less than 0.001, 0.01, 0.05, or 0.1, respectively. HINT-8, Health-related Quality of Life Instrument with eight items.

**Fig 2:**
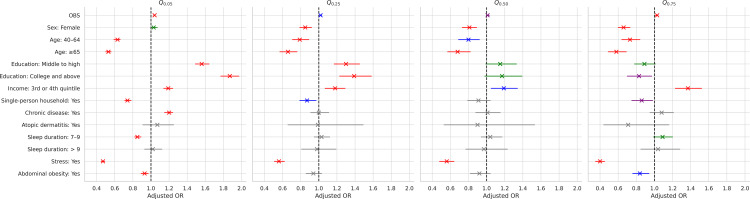
Estimated odds ratios and their 95% confidence intervals for regression parameters based on the logistic quantile regression model. Covariates are presented in the same order as Table 3, and colors are used to enhance interpretability according to p-value thresholds: red (p < 0.001), blue (p < 0.01), purple (p < 0.05), green (p < 0.1), and gray (not significant).

To enhance interpretability, we present a forest plot (Fig 2) that visualizes the estimated odds ratios and their 95% confidence intervals from Table 3. This figure effectively illustrates effect sizes across quantiles of the HINT-8 index, with color-coding to denote varying levels of statistical significance.

Socio-demographic covariates, including age, gender, education, and income, were statistically significant at the 0.1 significance level. Specifically, the median HINT-8 indices for the 40–64 and 19–39 age groups were 1.46 (*p* < 0.001) and 1.17 (*p* = 0.002) times higher, respectively, than for individuals aged 65 years and older. The median HINT-8 index was also 1.24 times higher in men than in women (p < 0.001). Additionally, the index was 1.17 times higher for college graduates (*p* = 0.082) and 1.15 times higher for middle to high school graduates (**p* *= 0.070) compared to individuals with an elementary education or less. Furthermore, the index was 1.19 times higher among individuals in the first to second income quintile than among those in the third to fourth quintile (*p* = 0.001). Participants who reported experiencing stress had a median HINT-8 index 1.80 times lower than those who did not (*p* < 0.001).

In contrast, the presence of AD, CDs, and sleep duration showed no significant association with the HINT-8 index. Specifically, individuals without CDs had a median HINT-8 index 1.03 times higher than those with CDs, while individuals without AD had an index 1.06 times higher than those with AD. Regarding sleep duration, participants who slept 7–9 h and those who slept more than 9 h had median HINT-8 indices 1.01 and 1.16 times higher, respectively, than those who slept less than 7 h. Additionally, individuals in multiple-person households had a median HINT-8 index 1.10 times higher than those in single-person households, and individuals without obesity had a median index 1.08 times higher than those with obesity; however, these results were not statistically significant.

Finally, a summary of the minimum, median, and maximum of the estimated quantiles or expected means is provided for each quantile.

**Fig 3:**
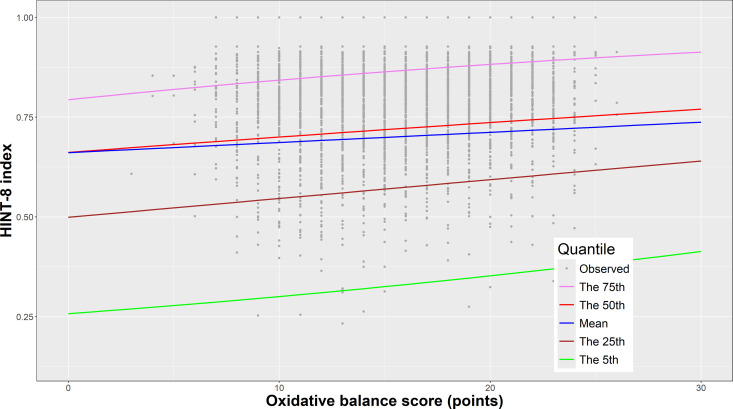
Estimated quantiles of the Health-related Quality of Life Instrument with eight items (HINT-8) index plotted against the oxidative balance scores (OBS), based on the logistic quantile regression model. The graph includes the 5th percentile (green line), 25th percentile (brown line), 50th percentile or median (red line), and the 75th percentile (purple line), along with the estimated mean (blue line).

Fig 3 illustrates how the estimated quantiles of the HINT-8 index vary with the OBS. For each covariate in Table 2, the category with the lowest mean HINT-8 index was used to fix the values of the other covariates, except for the OBS, which was set at regular intervals between 0 and 30. The mean HINT-8 index was slightly higher than the median, likely due to the ceiling effect of the HINT-8. A steeper slope was observed for smaller quantiles.

## Discussion

Using quantile regression, this study analyzed the relationship between OS and HRQoL in the general adult population. Significant differences were observed between men and women in the 15 components of the OBS, with all but retinol and physical activity showing statistically significant variation [17]. Men exhibited higher scores for antioxidant intake, including vitamins, indicating a more favorable oxidative balance from the antioxidant perspective. In contrast, women demonstrated lower intake of pro-oxidants—such as SFA, PUFA, alcohol, and tobacco—suggesting healthier lifestyle behaviors that reduce oxidative stress exposure. These findings indicate that while men benefit from greater antioxidant intake, women experience reduced oxidative stress through lower pro-oxidant exposure. Such differences may stem from underlying biological factors, sociocultural influences on health awareness and behavior, or a combination of both. Further research is warranted to clarify the relative contributions of these determinants. From a public health perspective, improving OBS for effective health management may require sex-specific strategies, such as promoting antioxidant-rich diets for women and encouraging reductions in pro-oxidant exposure for men.

Socio-demographic factors, such as age and household type, and health-related factors, including AD, CDs, and sleep duration, were found to differ in relation to the OBS. Additionally, we considered stress and abdominal obesity but acknowledge that other factors, such as mental health and diet quality, should be explored in future studies. Specifically, individuals aged ≥ 65 years had higher OBSs than those in younger age groups [39]. This result may be attributed to increased health awareness as individuals experience changes in health status with aging, leading to lifestyle modifications and increased consumption of dietary supplements [40]. Additionally, individuals in multi-person households had higher OBSs compared to those living alone. This finding is consistent with the study by Song et al. [41] and suggests that living with family may be advantageous for maintaining health. Participants with CDs had higher OBSs than those without CDs. These results differ from those of a systematic review, which reported that OBS is associated with a reduced risk of CDs, chronic kidney disease, colorectal adenomas, and various cancers [24]. Additionally, related studies have shown that higher OBSs were linked to lower hypertension in a community-based prospective cohort study [22], and that glycemic control improved with increasing OBSs among individuals with type 2 diabetes mellitus [23]. The discrepancy between the results of this study and previous research may be due to the inclusion of five diseases as chronic conditions in this study, which may have led to mixed results regarding the association between OBS and CDs, thereby reducing the clarity of the findings. In particular, osteoarthritis, which was included in this study, has also been shown in previous research to have no association with OBS [12]. Therefore, future research should classify chronic diseases individually to better elucidate their relationship with OBS. This study investigated the independent association between OBS and AD and found that individuals without AD had higher OBSs compared to those with the condition. In a systematic review of the association between OS and AD, the results of individual studies were inconsistent. However, the findings generally suggest that OS plays a critical role in AD [42]. AD is a common, multifactorial, chronic, relapsing inflammatory skin disease, and further research is needed to explore the overlooked aspects of OS in its pathogenesis.

This study found that individuals who slept less than 7 h had a higher OBS compared to those who had sufficient or excessive sleep. This result differs from previous research [43,44] and does not align with findings that sleep deprivation increases ROS levels, activating various response pathways [45], and that OS is a key factor in regulating sleep homeostasis [46,47]. Meanwhile, metabolomic studies have reported that sleep deprivation increases the production of ROS, whereas low-intensity exercise significantly reduces ROS levels [48]. Given the possibility that factors such as low-intensity exercise, which counteract the increase in ROS caused by insufficient sleep, may have influenced the findings of this study, further research is needed to identify mediating variables in the relationship between sleep and OS.

In this study, the OBS was found to improve HRQoL across all quantiles, supporting findings that the OBS can positively influence the overall health of the general adult population [12,24]. Additionally, Fig 2 shows that the slope of the increase in HRQoL with respect to OBS differs across quantiles. Notably, the slopes were observed at the 25th and 75th percentiles, indicating that strategies aimed at increasing OBS may be more effective among individuals with lower or higher levels of HRQoL. These findings suggest that oxidative stress may adversely affect HRQoL through multiple biological mechanisms. Specifically, oxidative stress has been shown to contribute to chronic inflammation, oxidative damage to cellular components, and dysregulation of neuroendocrine systems [3,4]. Excessive accumulation of ROS can activate pro-inflammatory signaling pathways, leading to systemic inflammation that is closely associated with impaired physical and mental health outcomes [6,42]. In addition, oxidative damage to essential biomolecules such as lipids, proteins, and DNA may impair cellular function, while disruptions in redox-sensitive signaling pathways may alter neuroendocrine homeostasis—affecting the regulation of stress hormones, sleep, and other physiological processes integral to HRQoL [4,45]. Meanwhile, in the LQM, gender, age, and income variables showed statistical significance across all quantiles. However, single-person households and sleep duration exhibited varying effects across quantiles, suggesting that these findings could inform the prioritization of target groups and the implementation of individualized, tailored health interventions aimed at improving HRQoL.

In light of these findings, the OBS may serve not only as a research measure but also as a practical tool for public health and clinical applications. In clinical settings, OBS could support personalized health counseling by identifying imbalances between antioxidant intake and pro-oxidant exposure, guiding dietary and lifestyle modifications. At the community level, OBS could be used to monitor oxidative balance across population subgroups and inform targeted public health initiatives—such as nutrition education, behavioral interventions, and chronic disease prevention programs.

This study had some limitations. First, given the cross-sectional nature of this study, it was not possible to confirm a causal relationship between OBS and HRQoL. Further longitudinal or intervention-based research is necessary to establish the causal link between these variables.

Second, although we attempted to review all OBS components in the KNHANES, we could not include them all. Third, the dietary components of the OBS were based on 24-h self-reported data, which might introduce measurement error and bias, potentially reducing accuracy by failing to capture daily dietary variations. Fourth, due to the absence of a statistical inference method for LQM that accounts for individual weights, we were unable to incorporate individual weights, unlike the Rao−Scott chi-square test and mean comparisons using t-tests or F-tests. Finally, as this study was conducted in Korea, the findings may not be generalizable to other populations. Future research should examine whether similar associations hold in diverse groups.

## Conclusion

This study confirmed a significant relationship between the OBS and HRQoL. To improve OBS, it is essential to increase the intake of antioxidant-rich nutrients, limit pro-oxidant intake, promote physical activity, and encourage smoking cessation and moderate alcohol consumption. Additionally, socio-demographic factors such as age, gender, education, and income were found to be associated with HRQoL. The findings suggest that OBS can serve as an effective indicator for health management interventions. This study identified modifiable factors for OS regulation and examined socio-demographic factors associated with HRQoL, providing a foundation for developing tailored health intervention strategies for diverse populations. Addressing these limitations in future research will enhance the rigor and practical applicability of OBS in real-world health programs.
